# 14 Bacterial Dysbiosis and Decreased SCFAs Following Alcohol Intoxication and Burn Injury

**DOI:** 10.1093/jbcr/irae036.014

**Published:** 2024-04-17

**Authors:** Mary Grace Murray, Caroline Herrnreiter, Xiaoling Li, Mashkoor A Choudhry, Marisa Luck, Abigail Cannon

**Affiliations:** Northwestern University Chicago, Lombard, Illinois; Loyola University Chicago, Maywood, Illinois; Loyola University Chicago, Chicago, Illinois; Loyola University Chicago, Oak Park, Illinois; Northwestern University Chicago, Lombard, Illinois; Loyola University Chicago, Maywood, Illinois; Loyola University Chicago, Chicago, Illinois; Loyola University Chicago, Oak Park, Illinois; Northwestern University Chicago, Lombard, Illinois; Loyola University Chicago, Maywood, Illinois; Loyola University Chicago, Chicago, Illinois; Loyola University Chicago, Oak Park, Illinois; Northwestern University Chicago, Lombard, Illinois; Loyola University Chicago, Maywood, Illinois; Loyola University Chicago, Chicago, Illinois; Loyola University Chicago, Oak Park, Illinois; Northwestern University Chicago, Lombard, Illinois; Loyola University Chicago, Maywood, Illinois; Loyola University Chicago, Chicago, Illinois; Loyola University Chicago, Oak Park, Illinois; Northwestern University Chicago, Lombard, Illinois; Loyola University Chicago, Maywood, Illinois; Loyola University Chicago, Chicago, Illinois; Loyola University Chicago, Oak Park, Illinois

## Abstract

**Introduction:**

Ethanol intoxication seen in over 50% of burn patients is associated with more adverse outcomes, longer hospitalization, and increased mortality. Ethanol is known to exacerbate the intestinal damage and gut leakiness seen in burn and is associated with a significant increase in bacterial translocation which may contribute to SIRS and bacteremia. Short chain fatty acids (SCFAs) are imperative bacterial metabolites that provide a major source of energy for the intestinal epithelium and aid in maintenance of the intestinal epithelial barrier. In this study we aim to identify whether ethanol and burn injury influences the bacterial composition and SCFA contents of the GI tract of ethanol burn mice in hopes of identifying potential targets for therapeutic intervention.

**Methods:**

Male and female mice were gavaged with either EtOH or water. 12.5% TBSA was exposed and burn animals were immersed in 85°C water bath for 7s or a 37°C water bath for sham animals. Small intestinal (SI) and cecum fecal contents were collected. DNA was isolated and 16S rRNA gene sequencing was performed and analyzed with QIIME. Microbial composition was analyzed by UniFrac PCA and a pairwise permanova analysis. Difference between medians for bacterial populations were calculated with Mann-Whitney U test.

**Results:**

Ethanol burn (EB) versus sham vehicle (SV) samples exhibited distinct microbial populations in the SI and cecum. Male and female mice showed consistent changes in the relative abundance of bacterial phyla following EB in the SI and cecum. The pathogenic Enterococcaceae (p < 0.01) and Enterobacteriaceae (p < 0.01) families including the species of Shigella (p < 0.05), were increased following EB while SCFA producing bacteria Akkermansia (p < 0.05) and Roseburia (p < 0.05) were decreased. There was a decreasing trend in the concentration of total SCFAs following EB (p = 0.06), with the most significantly reduced SCFA being butyrate (p < 0.001).

**Conclusions:**

Taken together these findings suggest bacterial dysbiosis following ethanol and burn injury with increases in pathogenic bacteria and decreases in SCFA producing commensal bacteria. Decreased SCFAs are known to contribute to gut leakiness and disruption of the integrity of the GI barrier. Roseburia is a known butyrate producer, and Akkermansia is known to produce propionate and acetate.

**Applicability of Research to Practice:**

Understanding the perturbations within the gut microbiome following ethanol binge and burn gives us insights into failures of the GI tract barrier that may contribute to SIRS and bacteremia. Now that we have identified the reduction in potentially beneficial bacterial species, this reveals a mechanism of dysbiosis within the microbiome that may be targetable with probiotic therapies such as Akkermansia or Roseburia in hopes of ameliorating the gut leakiness and subsequent bacterial translocation from the GI tract observed in burn.

